# P-1404. Trends and Disparities in Viral Load Suppression Among People Living with HIV in Three Texas Cities: A Decade-Long Retrospective Study

**DOI:** 10.1093/ofid/ofae631.1579

**Published:** 2025-01-29

**Authors:** Angie Garcia, A L exander P Radunsky, Nicole Naiman, Lucia Cabrejos, Barbara S Taylor, Karen J Vigil, Mamta K Jain

**Affiliations:** UT Southwestern Medical Center, Dallas, Texas; Ut Southwestern, Dallas, Texas; UT Southwestern Medical Center, Dallas, Texas; UT Southwestern Medical Center, Dallas, Texas; University of Texas Health Science Center San Antonio, San Antonio, TX; UT Health Science Center, Houston, TX; UT Southwestern Medical Center, Dallas, Texas

## Abstract

**Background:**

HIV/AIDS remains a significant public health challenge in the United States, especially in states with diverse populations such as Texas. This study examines viral load trends among people living with HIV in Houston, Dallas, and San Antonio over ten years. The aim is to investigate variations in viral load suppression among individuals living with HIV across different cities, considering demographic variables like age, gender, race/ethnicity, and socioeconomic status.

**Figure:**
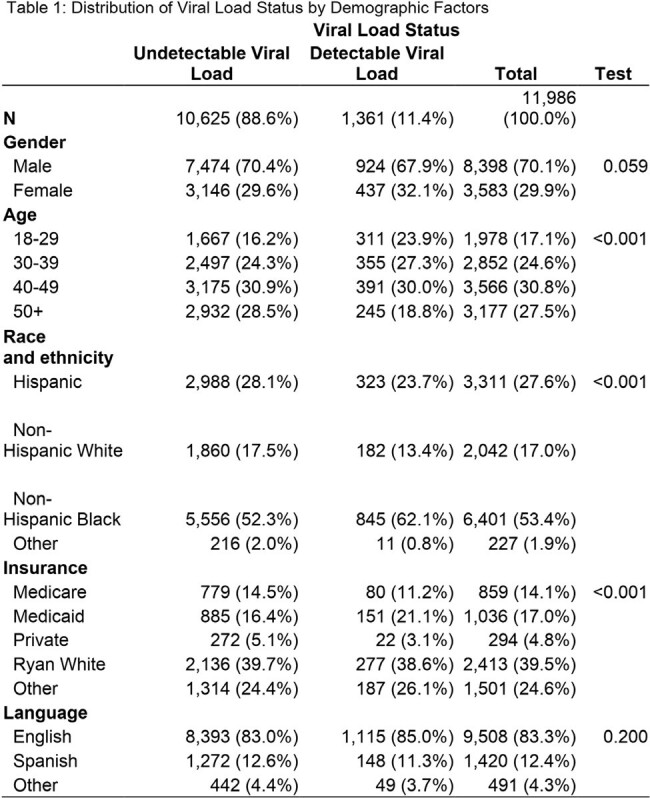

**Methods:**

A retrospective study was conducted on people with HIV (PWH) from 2009 to 2019. Demographic data, clinical variables, and viral suppression status were analyzed across different groups using time-to-event analysis.

**Figure:**
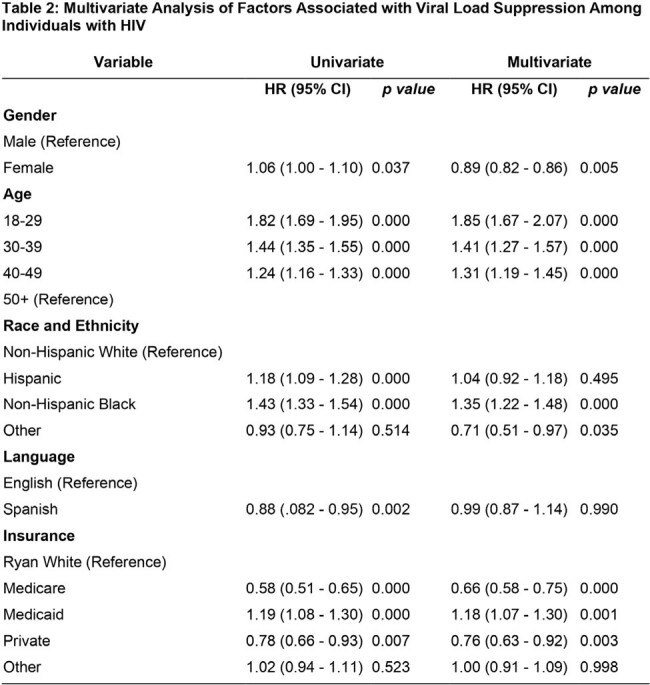

**Results:**

The analysis revealed that being female was linked to a reduced hazard ratio for detectable viral load (HR: 0.89, 95% CI: 0.82 - 0., p = 0.005) after adjusting for other demographic factors (table 2). Additionally, younger age groups (18-29, 30-39, and 40-49) exhibited significantly higher hazard ratios compared to those aged 50 and above, indicating a higher risk of detectable viral load. Race/ethnicity emerged as a significant factor, with Non-Hispanic Black individuals displaying a higher hazard ratio compared to Non-Hispanic White individuals (HR: 1.35, 95% CI: 1.22 - 1.48, p < 0.001). Furthermore, Medicaid-insured individuals demonstrated higher risk of detectable viral load, consistent with both univariate and multivariate analyses. However, language preference did not significantly influence viral load detection status, as evidenced by the absence of a significant difference between English and Spanish speakers in the multivariate analysis.

**Conclusion:**

Our research investigates disparities in achieving viral load suppression among individuals with HIV in Texas. Younger people seem to encounter challenges in maintaining viral suppression compared to older age groups. The influence of social determinants of health is highlighted by racial/ethnic disparities. While our study did not find a significant impact of language barriers on viral load detection, further exploration is needed to better comprehend these results.

**Disclosures:**

**Karen J. Vigil, MD**, Gilead: speaker|theratechnologies: research|ViiV: Advisor/Consultant **Mamta K. Jain, MD, MPH**, Abbvie: Grant/Research Support|Gilead Sciences: Grant/Research Support|Laurent: Grant/Research Support

